# Laparoscopic Removal of a Large Spermatic Cord Lipoma During Bilateral Transabdominal Preperitoneal Inguinal Hernia Repair: A Case Report and Narrative Review of the Literature

**DOI:** 10.7759/cureus.109615

**Published:** 2026-05-25

**Authors:** Kostas Tepelenis, Maria Alexandra Kefala, Stasini Gkorezi, Konstantinos-Marios Papigkiotis, Karolina Kokkoni, Gabriel Dahan, Marios Valakostas, Erasmia Rapti, Georgios Anthis, Angeliki Siafaka, George Mpourazanis

**Affiliations:** 1 Surgery, General Hospital of Ioannina G. Hatzikosta, Ioannina, GRC; 2 Paediatrics, General Hospital of Ioannina G. Hatzikosta, Ioannina, GRC; 3 General Surgery, Hygeia Hospital / University of Nicosia, Ioannina, GRC; 4 Otolaryngology - Head and Neck Surgery, General Hospital of Ioannina G. Hatzikosta, Ioannina, GRC; 5 General Surgery, General Hospital of Ioannina G. Hatzikosta, Ioannina, GRC; 6 Obstetrics and Gynaecology, General Hospital of Ioannina G. Hatzikosta, Ioannina, GRC

**Keywords:** inguinal hernia, lipoma, pseudo-recurrence, round ligament lipoma, spermatic cord lipoma

## Abstract

Surgeons should be mindful of spermatic cord lipomas (or round ligament lipomas in women), as their presence may be overlooked during inguinal hernia repairs, leading to early pseudo-recurrence. These tumors can occur alongside an indirect hernia sac or on their own. Lipomatous lesions of the inguinal canal may occur as spermatic cord lipomas in men or round ligament lipomas in women. The incidence of these tumors varies, but they are more common in men*. *However, the estimated prevalence of these tumors that are not associated with an indirect hernia sac is quite low. It is important to note that these lesions are not true lipomas; rather, they originate from pre-peritoneal fat, travel through the inguinal canal, and may reach the testicles via the superficial inguinal ring. There is no consensus in the literature regarding the size threshold that categorizes these tumors as "large" or "giant." Clinically, preoperative diagnosis may be difficult, although spermatic cord lipoma can sometimes be suspected on physical examination, as they often present with symptoms similar to those of inguinal hernias. One useful method for identifying spermatic cord lipomas preoperatively is ultrasonography. Any discrepancy between preoperative findings and intraoperative observations should raise suspicion about the potential presence of a spermatic cord lipoma. All identified lipomas should either be excised or at least reduced, unless their presence obstructs the placement of the preperitoneal mesh. In this case, a 47-year-old male underwent a laparoscopic bilateral inguinal hernia repair using the transabdominal preperitoneal (TAPP) method. During the dissection of the left indirect inguinal hernia sac, a lipomatous mass measuring 10 x 5 cm was discovered, originating from the preperitoneal fat, and was subsequently excised. This report summarizes the available data on spermatic cord lipomas.

## Introduction

The most common benign tumors found in the inguinal canal are lipomas [1], often discovered incidentally during open or laparoscopic inguinal hernia repairs. These tumors are known by several names, with "spermatic cord lipoma" being the preferred term for males and "round ligament lipoma" for females [2]. It is important to note that a spermatic cord lipoma is not a true lipoma; rather, it originates from preperitoneal fat, is located within the internal spermatic fascia of the spermatic cord, and can extend through the inguinal canal down to the testicles. By contrast, a true lipoma is composed of adipose tissue, is confined to the inguinal canal, and does not connect with preperitoneal fatty tissue [3].

There is no consensus in the literature regarding the size classification of spermatic cord lipomas. Generally, a lipoma greater than 10 cm is considered "large," while those exceeding 15 cm are classified as "giant" [4,5]. Spermatic cord lipomas may accompany an indirect hernia sac or occur independently. Distinguishing between spermatic cord lipomas and inguinal hernias before surgery can be complex, as their clinical manifestations are similar. Any discrepancies between preoperative and intraoperative findings should raise suspicion about the presence of a spermatic cord lipoma. A useful technique during laparoscopic inguinal hernia repair for identifying a spermatic cord lipoma is applying external compression to the superficial inguinal ring [4,6].

Spermatic cord lipomas should generally be reduced or excised when identified during inguinal hernia repair, particularly if they contribute to symptoms or interfere with mesh placement. However, in selected cases, a lipoma may be left in situ provided the finding is documented, and the patient is appropriately informed postoperatively regarding the possibility of persistent bulging or pseudo-recurrence. However, removing these tumors during surgery, whether open or laparoscopic, can be challenging due to their proximity to the structures of the spermatic cord, which increases the risk of injury to these structures [3,4]. This report discusses a case involving a 47-year-old male who underwent laparoscopic bilateral inguinal hernia repair using the transabdominal preperitoneal (TAPP) method. During the dissection of the left indirect hernia sac, a large spermatic cord lipoma measuring 10 cm x 5 cm was observed and subsequently removed.

## Case presentation

A 47-year-old male patient was referred to the surgical outpatient clinic for laparoscopic repair of a bilateral inguinal hernia. He has a medical history of Grade I obesity (BMI of 34 kg/m²) and reports no previous surgical history. In addition, he did not smoke and consumed alcohol occasionally. The patient noticed a soft mass in both the right and left inguinal regions two months ago. Following a visit to a local hospital, he was diagnosed with a bilateral inguinal hernia.

Clinical examination revealed bilateral reducible inguinal hernias, with greater prominence on the left side. In our clinic, routine abdominal ultrasounds or computed tomography scans are not typically conducted for inguinal hernia evaluations unless specific circumstances warrant them. The patient was scheduled for a TAPP inguinal hernia repair. Three months later, the patient was admitted to the surgical department for the laparoscopic repair of his bilateral inguinal hernia. Laboratory blood tests were within normal limits (Table 1).

**Table 1:** Laboratory test results WBC, white blood cells; NEUT, neutrophils; LYMPH, lymphocytes; MONO, monocytes; EO, eosinophils; BASO, basophils; RBC, red blood cells; HGB, hemoglobin; HCT, hematocrit; MCV, mean corpuscular volume; MCH, mean corpuscular hemoglobin; MCHC, mean corpuscular hemoglobin concentration; PLT, platelets; PT, prothrombin time; INR, international normalized ratio; aPTT, activated partial thromboplastin time; GLC, glucose; URE, urea; CRE, creatinine; K+, potassium; Na+, sodium; MG, magnesium; TBL, total bilirubin; DBL, direct bilirubin; TPR, total protein; ALB, albumin; ALP, alkaline phosphatase; AST, aspartate aminotransferase; ALT, alanine aminotransferase; γGT, gamma-glutamyl transferase; LDH, lactate dehydrogenase; CK, creatine kinase; AMY, amylase; CA, calcium; CHE, cholinesterase

Test	Result	Reference range
WBC	5.55 k/μL	4-11 k/μL
NEUT	58.4%	40-75%
LYMPH	33.9%	20-45%
MONO	5.8%	2-10%
EO	1.4%	1-6%
BASO	0.5%	0.2-1%
RBC	5.19 M/ μL	3.8-6 M/ μL
HGB	15.1 g/dl	11.8-17.8 g/dl
HT	44.2%	36-52%
MCV	85.2 fl	80-96 fl
MCH	29.1 pg	26-32 pg
MCHC	34.2 g/dl	32-36 g/dl
PLT	228 k/μL	140-450 k/μL
PT	11.3 sec	-
INR	0.94	1-1.3
aPTT	32.4 sec	25-38 sec
GLC	70 mg/dl	70-115 mg/dl
URE	41 mg/dl	10-50 mg/dl
CRE	1.24 mg/dl	0.8-1.4 mg/dl
K	4.4 mmol/L	3.5-5.1 mmol/L
Na	143 mmol/L	136-146 mmol/L
MG	1.65 mEq/L	1.3-2.1 mEq/L
TBL	1.47 mg/dl	0.1-1.3 mg/dl
DBL	0.37 mg/dl	0-0.5 mg/dl
TPR	7.2 g/dl	6.2-8.4 g/dl
ALB	4.4 g/dl	3.5-5.1 g/dl
ALP	49 IU/L	35-125 IU/L
AST	24 IU/L	5-40 IU/L
ALT	38 IU/L	5-40 IU/L
γGT	29 IU/L	8-45 IU/L
LDH	166 IU/L	120-230 IU/L
CK	189 IU/L	0-220 IU/L
AMY	60 IU/L	28-100 IU/L
CA	9.3 mg/dl	8.2-10.5 mg/dl
CHE	8597 IU/L	4600-11500 IU/L

**Figure 1:**
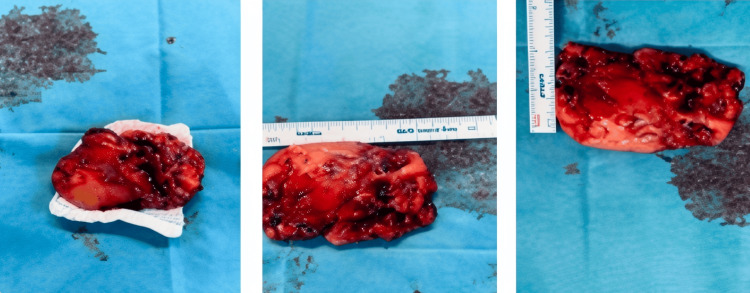
Spermatic cord lipoma measuring 10 x 5 cm

The surgical procedure employed a three-trocar approach. The pneumoperitoneum was established using the open-entry technique known as Hasson’s technique, and a 10 mm optical port was inserted in the supraumbilical region. Two additional 5 mm working ports were placed under direct vision on the left and right flanks, approximately 7 cm from the umbilicus. A right direct inguinal hernia and a left indirect inguinal hernia were identified during the procedure. The right inguinal hernia was addressed first. The retroperitoneal space was developed using a combination of blunt and sharp dissection with scissors. Subsequently, the left inguinal hernia was approached in a manner similar to that of the right side. During the dissection of the left hernia sac, a sizeable lipomatous mass was observed in the spermatic cord, connected to the preperitoneal fatty tissue via the deep inguinal ring. This mass was carefully dissected away from the overlying peritoneum and the structures of the spermatic cord before being excised. The right and left hernia repair sites were each covered with a 10 x 15 cm 3D polypropylene heavyweight mesh (Dextile anatomical mesh by Medtronic). Each mesh was secured to Cooper’s ligament and the ipsilateral rectus muscle, lateral to the inferior epigastric vessels, ensuring that the cutaneous nerves were avoided, using absorbable tackers. The peritoneal flap was then closed over each mesh with absorbable tackers. The specimen, which included the lipomatous mass measuring 10 x 5 cm (Figure 1), was extracted through an extended supraumbilical port, which was subsequently closed.

The postoperative period was uneventful, and the patient was discharged the following morning. The histopathology report confirmed the diagnosis of an encapsulated lipoma without malignant characteristics.

## Discussion

Lipomas found in the inguinal canal are often discovered incidentally during surgical repairs of inguinal hernias [2,4]. Various terms have been used in the literature to refer to these tumors, including “inguinal cord lipomas”, “lipomas of the cord”, “spermatic cord lipomas”, and “round ligament lipomas” [2]. It is important to differentiate that a spermatic cord lipoma is not a true lipoma of the inguinal canal; rather, it consists of preperitoneal fatty tissue that extends through the superficial inguinal ring down to the testicle. This lipoma is situated within the internal spermatic fascia of the spermatic cord and is in close proximity to its associated structures, which include the vas deferens, testicular artery, deferential artery, cremasteric artery, pampiniform plexus, genital branch of the genitofemoral nerve, ilioinguinal nerve, and lymphatic vessels. By contrast, a true lipoma of the spermatic cord is a benign tumor made up of adipose tissue that is confined to the inguinal canal and does not connect with preperitoneal fat [3]. The distinction between a true lipoma and a spermatic cord lipoma is based on two key characteristics of true lipomas: they do not connect with preperitoneal fat and show no vascularization on color Doppler imaging [7,8].

Spermatic cord lipomas are the most common benign tumor found in the inguinal canal [1,9]. They can occur either alongside an indirect hernia sac or independently, without an indirect hernia sac present [6]. Their incidence, both with and without an indirect hernia sac, ranges from 20% to 75% among all inguinal hernia repairs [3]. Higher incidences have been noted in both post-mortem and clinical studies. For instance, in a cadaveric study conducted by Heller et al., spermatic cord lipomas were identified in 75% of the 36 body halves examined [10]. Similarly, Carilli et al. evaluated 128 consecutive patients with 139 indirect inguinal hernias undergoing open repair, finding that 72.5% of these patients had an associated spermatic cord lipoma [11]. By contrast, the prevalence of spermatic cord lipomas in the absence of an indirect hernia sac is only between 1% and 8% [3].

The incidence of round ligament lipomas is significantly lower, estimated to occur in 12.5-36% of all inguinal hernia repairs in women [2,12]. However, the prevalence of round ligament lipomas without an indirect hernia sac is only 1.4% among female inguinal hernia repairs [13]. This difference in incidence between spermatic cord lipomas and round ligament lipomas can be attributed to the structural differences; the round ligament comprises a band of adipose tissue with accompanying blood vessels and lacks the inner matrix of adipose tissue derived from the preperitoneal tissue that is present in the spermatic cord [3].

The cause of spermatic cord lipomas remains unknown, although a theory suggests a developmental origin [5,14]. These lipomas typically affect individuals in their 40s or 50s, show a male predominance, and are more commonly found on the left side [6,14]. They are classified as lateral hernia L1 (≤1 finger) according to the European Hernia Society classification system [15]. In the literature, there is no consensus on the definitions of “large” and “giant” lipomas; however, a lipoma greater than 10 cm is generally considered large, while one exceeding 15 cm is classified as giant [4,5]. Differentiating spermatic cord lipomas from inguinal hernias can be challenging due to their similar clinical presentation and physical examination findings [4,6,14,16].

It is essential to remove all spermatic cord lipomas or, at the very least, reduce their size, provided they do not obstruct the placement of a pre-peritoneal mesh. This is important regardless of whether they are associated with a hernia sac, as failing to address them may lead to early pseudo-recurrence. Any discrepancy between the preoperative clinical bulge and the findings observed during surgery should raise suspicion of the presence of a spermatic cord lipoma. Some authors suggest applying external pressure at the superficial inguinal ring to help identify a spermatic cord lipoma during laparoscopic inguinal hernia repair. When dealing with these lipomas, it is advisable to use blunt dissection techniques while preserving the surrounding fatty tissue. The fatty tissue associated with spermatic cord lipomas originates from the preperitoneal space and receives its blood supply from there as well. This tissue can be either reduced or resected; however, resection is only necessary if it obstructs the placement of the post-reduction mesh. There are no documented cases in the literature indicating that reducing spermatic cord lipomas poses a risk of secondary infection due to devascularized fatty tissue, which supports the theory that these lipomas receive their blood supply from the preperitoneal space [3,4,15].

## Conclusions

Spermatic cord lipomas are the most common benign tumors found in the inguinal canal and are typically discovered incidentally during surgical repair of inguinal hernias. They are not true lipomas, as they originate from preperitoneal fat, extend into the spermatic cord located within the internal spermatic fascia, and can reach the testicles through the superficial inguinal canal. Spermatic cord lipomas may occur individually or accompany an indirect hernia. Differentiating a spermatic cord lipoma from an indirect inguinal hernia preoperatively is challenging, as both present similar symptoms and signs. Therefore, all spermatic cord lipomas should be excised or at least reduced, as they can contribute to early pseudo-recurrence. Any inconsistency between the preoperative bulging and intraoperative findings should heighten suspicion regarding the presence of a spermatic cord lipoma. It is crucial for all surgeons to be aware of these common benign tumors of the inguinal canal.
